# Feeding Patterns, Mother-Child Dietary Diversity and Prevalence of Malnutrition Among Under-Five Children in Lebanon: A Cross-Sectional Study Based on Retrospective Recall

**DOI:** 10.3389/fnut.2022.815000

**Published:** 2022-02-28

**Authors:** Huguette Abi Khalil, Mariam Hawi, Maha Hoteit

**Affiliations:** ^1^Faculty of Public Health, Lebanese University, Beirut, Lebanon; ^2^PHENOL Research Group (Public Health Nutrition Program-Lebanon), Faculty of Public Health, Lebanese University, Beirut, Lebanon; ^3^Lebanese University Nutrition Surveillance Center (LUNSC), Lebanese Food Drugs and Chemical Administrations, Lebanese University, Beirut, Lebanon

**Keywords:** dietary diversity, maternal, malnutrition, children under five, overweight, underweight

## Abstract

**Background:**

Despite demonstrated benefits, most countries fall short of meeting international targets for breastfeeding patterns, optimal complementary feeding, mother-children's quality diet, and malnutrition among under-five children.

**Rationale:**

Since mothers usually play the most vital role in the healthcare of their children, research is needed to illuminate maternal factors that might promote a child's health and nutritional status.

**Aim:**

The purpose of this study is to retrospectively (1) examine the under-five children's (0–59 months) feeding habits including exclusive breastfeeding, exclusive bottle feeding, continued breastfeeding, and complementary feedings, (2) investigate the mother-child's dietary diversity, and (3) identify any factors that cause less optimal nutrition due to a lack of food diversity in children aged 6 to 59 months. Moreover, (4) the prevalence of wasting, stunting, underweight, and overweight in the under-five offspring living in households located in the main two provinces in Lebanon (Beirut and Mount Lebanon) was determined.

**Methods:**

The data for this analysis were collected from a representative sample of 384 households [384 mothers (21–49 years old) and children (0–59 months)] between February 2019 and June 2019. A questionnaire was used to inquire mothers of children ages 0–23 months about exclusive breastfeeding (EBF), continuous breastfeeding (CBF), exclusive bottle feeding (EBOT), mixed feeding (MF), and complementary feeding patterns. Moreover, additional questions regarding dietary diversity were asked to mothers of children aged 6 to 59 months. This score was calculated based on the 24 h recall of the mother and her child's consumption of 7 food groups, during the 24 h prior to the survey. Moreover, stunting, wasting, overweight, and underweight were calculated using the z-score for height-for-age (HAZ), weight-for-height (WHZ), and weight-for-age (WAZ), respectively. Binary logistic regression was used to explore the dietary diversity among children (ages 6–59 months) adjusting for covariates at maternal and household levels.

**Results:**

Around 44% of children (0–59 months) had normal body weight. In addition, 9.3% were underweight (WAZ < -2SD to −3SD), 6.5% were at risk of being overweight, 24.45% were overweight, 9.3% were stunted (HAZ < -2SD to −3SD), and 6.25% (WHZ < -2SD to −3SD) were wasted. In total, among under-five children, the prevalence of EBF at 40 d and 6 months was 27 and 30%, respectively. The prevalence of CBF was 23%. Around 60% of mothers breastfed their offspring between 0 and 6 months and half of them introduced infant formula at earlier stages between 0–6 months. Furthermore, 78.4% of mothers introduced food to their children between 4 and 6 months (of which 40% before 6 months) and 62.5% of them introduced sugary drinks before 6 months. As for dietary diversity (DD), one out of two mothers and one out of three children (ages 6–59 months) had a low DD score (DDS) (46 and 32%, respectively). The children's and mother's DD were strongly found to be correlated (*p*-value = 0.034). Regression analysis showed that children's DD increased around 2 times [AOR = 1.7; 95% CI (1.042–2.914)] in context of high maternal DDS, and about 12 times [AOR = 11.7; 95% CI (1.2–111)] when a member of the highest-income households.

**Conclusions:**

Our findings demonstrated low rates of EBF and CBF, high prevalence of EBOT, and early introduction of complementary foods among children ages 0–59 months. Furthermore, for children ages 6–59 months, there was poor mother-child dietary diversity and a high prevalence of overweight and stunted children in the main two Lebanese provinces. This suggests the alarming need for continuous nutrition intervention to improve infant feeding patterns and dietary diversity to reduce the malnutrition rates.

## Introduction

It was long believed that malnutrition among children concerned only the underdeveloped countries; however, it also poses a serious challenge in developing countries (1). Childhood undernutrition contributes to 45% of mortality globally in children aged under 5 years (1). Malnutrition has been defined by the World Health Organization (WHO) as “deficiencies, excesses or imbalances in a person's energy intake or nutrient intake” (2). In addition, the term *malnutrition* refers to three broad groups which are under-nutrition, micronutrient-related malnutrition, and overweight/obesity (2). According to WHO, in 2020, globally, 149.2 million children under the age of 5 years were stunted (22%), 45.4 million were wasted (13.6%), and 38.9 million were overweight (5.7%), respectively (3). Chronic childhood malnutrition (stunting) remains a major challenge in the Eastern Mediterranean Region (EMR) including Lebanon (4). Millions of children under 5 years old in the region have had their growth stunted by chronic malnutrition, with serious lifelong consequences for their health and development (4). In Lebanon, the prevalence of stunting ranged between 16% in 2000 to 10.4% in 2020 (5). As for overweight and obesity, in 2018, the estimated weighted average prevalence among children under 5 years old was 8.42% in the EMR, and between 17.6 and 19.7% in Lebanon (5). EBF for under 6 months children, CBF for under 2 years children, appropriate complementary feeding practices, and adequate dietary diversity (DD) are needed at this particular age to ensure proper growth and development (2). The WHO and the United Nations International Children's Emergency Fund (UNICEF) recommend starting breastfeeding within 1 h of birth, EBF for the first 6 months of life, and introducing nutritionally adequate and safe complementary (solid) foods at 6 months with CBF for 2 years or longer (6). The WHO initially set a global target of 50% prevalence of exclusive breastfeeding by 2025 (6). Recently, it was updated to at least 70% prevalence by 2030 (6). Previous research has shown that proportion of exclusively breastfed children remains low in many lower- and middle-income countries (7). However, according to a recent geospatial analysis of EBF prevalence estimates from 2000 to 2018 across 94 low-middle-income countries (LMICS), the total prevalence of EBF increased from 27% to 39% across all countries (2000–2018) (8). It is estimated to be extrapolated to 43% by 2025 (8). Although this could be positive progress, it falls short of the 70% goal (8). Between conception and the child's second birthday, the first 1,000 days of life offer a golden chance for nutrition and lifestyle changes to shape the child's growth (9). Throughout this phase, optimal breastfeeding and complementary feeding practices are crucial for supporting fetal growth and development, maternal health, newborn and toddler growth (10), and for preventing a child's malnutrition and the development of non-communicable diseases (NCDs) (11). Despite the inconsistencies in the current guidelines regarding when to introduce complementary solid foods, all guidelines agree that complementary feeding should not be introduced before the age of 4 months (12). Although introducing complementary feeding earlier may contribute to more rapid weight gain during infancy and increased risk of childhood obesity in affluent populations (13), the introduction of complementary feeding before 4 months is common in many countries. For instance, the percentage of infants introduced to complementary solid foods before the age of 4 months was 37% in a birth cohort born in 2007 and 2008 in Northwest Italy (14), 30% across the UK in 2010 (15), and 40% among infants born between 2005 and 2007 participating in a national study in the United States (16). To the best of our knowledge, no study has reported the prevalence of introducing complementary feeding before 6 months in Lebanon. Lebanon, a middle-income country, has a low rate of breastfeeding (17). Misconceptions about breastfeeding among mothers and communities, a lack of professional lactation support, a failure to implement national policies that promote and protect breastfeeding practices, a lack of social support, particularly at the family level, and other socio-demographic factors are all barriers to breastfeeding (18). Diets high in starch-based staples but low in animal products, fresh fruits, or vegetables can result in micronutrient deficiencies, which can leave children vulnerable to undernutrition and its sequelae (2). According to the WHO and the UNICEF, the amount of various foods or food groups ingested over a certain reference period is referred to as DD (6, 19). It reflects household access to a variety of foods. DD serves as a proxy indicator for nutrient sufficiency in mothers and children's diets, particularly micronutrient adequacy, which is an important aspect of diet quality (6, 19). Abbreviated as DDS (dietary diversity score) is a tally of food types consumed over a specific time period, usually 24 h (6, 19). Women should consume more than or equal to five food groups in the prior 24 h to achieve the minimum requirement for a healthy diet, while children should consume more than or equal to four food groups (6, 19). At the household level, there are many causes that can contribute to malnutrition, including inadequate sanitation and water supply, low wealth and socioeconomic status, food insecurity, low status of women, poor caregiver education, inappropriate intra-household food allocation, inadequate quality foods, contaminated food and water, and infection. Other contributing factors include poor maternal nutrition and inadequate care, breastfeeding, or complementary feeding (4). There are many aspects of the wider context that can contribute, including food prices and trade policy, marketing regulations, political stability, poverty, access to healthcare, agriculture and food systems, education, society, and culture, as well as aspects of the environment (4). According to a recent meta-analysis, child age, child sex, complementary food, poor DD, diarrheal diseases, maternal education, maternal height, residential area, and socioeconomic status were significant risk factors for undernutrition (20). Thus, identification of potentially modifiable determinants of nutritional status in children is a priority in regions with a high burden of undernutrition.

Understanding mother/child DD and its influence on nutritional status in Lebanon may aid in the development of interventions to reduce malnutrition at a national level. To our knowledge, no study in Lebanon has reported the association between mother-child dietary diversity. Here we assess the under-five children's feeding habits (EBF, EBOT, CBF, and complementary feedings), the mother-child's DD, and any factors that cause less optimal nutrition due to a lack of food diversity in children aged 6 to 59 months. Moreover, the prevalence of wasting, stunting, underweight, and overweight in the under-five offspring living in households located in the main two provinces in Lebanon (Beirut and Mount Lebanon) was determined.

## Materials and Methods

### Sampling Strategy

A total of 400 mothers and children were included in the sample. Of these, 384 were included in the analyses. Thus, the data for this analysis were collected from a representative sample of 384 households located in the two main Lebanese provinces—Beirut and Mount Lebanon—and using a stratified cluster sampling design. Within each district, households were selected following a probability proportional to size approach. Housing units constituted the primary sampling unit in the two districts. A single population formula was used to determine the sample size. Accordingly, the formula for sample size determination used was *n* = [p (1-p)] ^*^ [(*Z*_∝/2_)^2^ / (*e*)^2^], where n denotes the sample size, *Z*_∝/2_ is the reliability coefficient of the standard error at 5% level of significance = 1.96, p represents the probability of under-five children who were unable to practice preventive measures against the diseases (50%, no previous study), and e refers to the level of standard error tolerated (5%) as stated by Hosmer and Lemeshow. Based on this formula, it was determined that the minimum acceptable sample size of 300 respondents would be sufficient to identify differences by respondent's characteristics.

### Data Collection

A total of 384 mothers (21–49 years old) and children (0–59 months) were included in this study between February 2019 and June 2019. Trained nutritionists administered the survey through face-to-face interviews with the mothers. A questionnaire was used to inquire mothers of children ages between 0 and 23 months about EBF, CBF (duration exceeding 6 months), EBOT, MF, and complementary feeding patterns. Moreover, additional questions regarding DD were asked to mothers of children ages 6 to 59 months. This score was calculated according to the WHO guidelines and based on the 24 h recall of the mother and her child's consumption of 7 food groups, during the 24 h prior to the survey (6, 19). The same food groups were used to compose both maternal and child DDS. Food groups considered were cereals/roots, vegetables, fruits, legumes/lentils, meat/fish/eggs, and milk/dairy products. If an individual consumes any quantity of any food group at least once per day, it was taken into count. Therefore, DDS was calculated without considering a minimum intake for the food group. To meet the minimum requirement for a healthy diet, mothers should obtain more than or equal to five while children above or equal to four food groups over the preceding 24 h (6, 19). Anthropometric measurements of the mother and child were collected. Stunting, wasting, overweight, and underweight were calculated using the z-score for height-for-age (HAZ), weight-for-height (WHZ), and weight-for-age (WAZ), respectively. We defined wasted, stunted, and underweight based on the z-scores of the 2006 WHO growth standards (6) as follows: wasted: weight for height z-score (WHZ) < -2SD to −3SD; stunted: height for age z-score (HAZ) < -2SD to −3SD; underweight: weight for age z-score (WAZ) < -2SD to −3SD. Binary logistic regression was used to explore the DD among children (6–59 months) adjusting for covariates at maternal and household levels.

### Eligibility Criteria and Definition of Feeding Practices

Mother-child dyads were eligible to participate in the study if the mother was Lebanese, aged 19–49 years. Children were eligible if they were five years old or younger, were born at term (gestational age between 37 and 42 weeks), and had no chronic medical conditions, inborn errors of metabolism, or physical malformations that interfered with feeding patterns and body composition.

Infant feeding practices were defined as follows:

1. Exclusive breastfeeding (EBF): The infant received breast milk from their mother or expressed breast milk and no other fluids or solids.
2. Mixed feeding: The infant received breast milk with formula milk and/or other fluids and/or solid food.
3. Exclusive bottle feeding (EBOT): The infant received formula milk with or without other fluids.

### Statistical Analyses

The statistical analysis of this study was performed using the Statistical Analysis Package for Social Science (SPSS, version 25) and the significance level was set at *p* < 0.05. Frequencies and descriptive statistics were performed for the general variables. Chi-square test was used to compare the categorical variables in the study as well as determine the prevalence of obesity, overweight, underweight, stunting, and wasting according to the child's age category. Chi-square was also used to assess the association between the mother's DDS and the child's DDS. In addition to that, independent *t-*test was conducted to compare the means between two unrelated groups (low child's DDS and high child's DDS) on the same continuous, dependent variable (mother's age). Binary logistic regression was used to explore the association between mothers' and children's DD adjusting for covariates at maternal and household levels with analyses represented as odds ratios and 95% confidence intervals.

### Ethics

Ethical approval was obtained from the Ethical Committee at the Lebanese University (#CU201907). The mother of the child signed a consent form, and the questionnaires were administered after the families agreed to participate in the study.

## Results

### Socio-Demographic Characteristics of Households and Study Participants: Mothers and Children

A total of 400 mothers and children were included in the sample. Of these, 384 were included in the analyses. The remaining 16 were excluded from the analysis because of missing data. Socio-demographic details are shown in Table 1. The mean age of mothers selected for this study was 32 ± 5 years old while the mean age for children was 23.8 ± 17.3 months. Half of the children were females. There were 200/384 children aged under 2 years and 183/384 children aged more than 2 years. Most mothers were educated and held a university degree (77%) and 60% of them were employed. More than half the households encompassed 1 child (52%) or 2 children (42%) aged less than 5 years and enclosed less than 5 members per household (60%). Around half the household's income ranged between 750,000 Lebanese Pound (LBP) and 3 million LBP (54%) (Table 1).

**Table 1:** Socio-demographic, weight status, and household characteristics of Lebanese infants and young children aged less than 5 years old.

	**Population characteristics N (%)**
Mother's age (years)	
Mean ± SD	32 ± 5
**Mother's age categories (years)** ***N*** **(%)**	
20–24 25–29 30–34 35–39 40–45	20 (5.2) 95 (24.7) 153 (39.8) 98 (25.5) 17 (4.4)
**Governorate of residency** ***N*** **(%)**	
Beirut Mount Lebanon	77 (20.1) 307 (79.9)
**Children at home** ** <5 years** ***N*** **(%)**	
1 child 2 children 3 children and more	199 (51.8) 161 (41.9) 24 (6.3)
**Household number** ***N*** **(%)**	
<5 members ≥5 members	229 (59.6) 155 (40.4)
**Mother's employment status** ***N*** **(%)**	
Employed Homemaker	232 (60.4) 152 (39.6)
**Mother's education level** ***N*** **(%)**	
Primary education Intermediate education Secondary education or technical diploma University degree and higher	3 (0.8) 26 (6.8) 61 (15.9) 294 (76.6)
**ousehold's income** ***N*** **(%)**	
<750,000 L.L. 750,000–1,500,000 L.L. 1,500,001–2,250,000 L.L. 2,250,001–3,000,000 L.L. >3,000,000 L.L	5 (1.3) 65 (16.9) 77 (20.1) 68 (17.7) 169 (44)
**Child age** ***N*** **(%)**	
0–6 months 6–59 months Mean	74 (19.3) 310 (80.7) 23.8 ± 17.3
**Child gender** ***N*** **(%)**	
Male Female	190 (49.5) 194 (50.5)
**Under-five children (*****n** **=*** **384)**	
Normal body weight Underweight (WAZ < −2SD to −3SD)^∧^ At risk for overweight Overweight Stunting (HAZ < −2SD to −3SD)^∧^ Wasting (WHZ < −2SD to −3SD)^∧^	170 (44.2) 36 (9.3) 25 (6.5) 93 (24.45) 36 (9.3) 24 (6.25)
**Under-two children (*****n** **=*** **200)**	
Normal body weight Underweight (WAZ < −2SD to −3SD)^∧^ Overweight Stunting (HAZ < −2 SD to −3SD)^∧^ Wasting (WHZ < −2 SD to −3SD)^∧^	97 (48.5) 14 (7) 40 (62.5) 25 (12.5) 24 (37.5)

∧ *Adapted from: http://ebook.ecog-obesity.eu/chapter-growth-charts-body-composition/world-health-organization-reference-curves/*.

### Prevalence of Stunting, Wasting, Overweight, and Obesity

Most of the children aged under 5 in this study had a normal body weight (44%). In addition, 9.3% were underweight (WAZ < -2SD to −3SD), 6.5% were at risk of being overweight, 24.45% were overweight, 9.3% were stunted (HAZ < -2SD to −3SD), and 6.25% (WHZ < -2SD to −3SD) were wasted. The prevalence of stunting, wasting, and overweight among under 2 children was 12.5, 37.5, and 62.5%, respectively (Table 1).

### Exclusive Breastfeeding, Exclusive Bottle Feeding, Continuous Breastfeeding, Mixed Feeding, and Complementary Feedings

#### Children 0–23 Months

The prevalence of EBF, EBOT, MF, and complementary feeding among children ages 0–23 months is shown in Table 2. The prevalence of EBF at 40 d was 26%, at 6 months was 25.5%, and the prevalence of CF (exceeding 6 months) was 12%. On the other hand, the prevalence of EBOT at birth was 52.8%, at 40 d was 80.5%, at 4 months was 7.1%, and at more than 6 months it was 19.3%. Moreover, the MF since birth was 18.5%. Around half the mothers breastfed their offspring between 0 and 6 months (51.5%) and introduced infant formula at earlier stages between 0 and 6 months. In addition, 68% of them introduced food to their children between 4 and 6 months (of which 40% before 6 months) and 53.5% of them introduced sugary drinks before 6 months (Table 2).

**Table 2:** Exclusive breastfeeding, continuous breastfeeding, exclusive bottle feeding, and complementary feedings among children aged 0–23 months (*n* = 200) and children aged 24–59 months (*n* = 184).

**Feeding patterns**	**0–23 months children *N* (%)**	**24–59 months *N* (%)**	**0–59 months *N* (%)**
**Currently breastfeeding**			
Yes No	72 (36) 128 (64)	6 (3.3) 177 (96.7)	78 (20) 306 (80)
**Duration of breastfeeding**			
0–40 d 40 d−6 months >6 months (continued breastfeeding)	52 (26) 51 (25.5) 24 (12)	53 (29) 64 (35) 62 (33.9)	105 (27.3) 115 (29.9) 87 (22.7)
**Exclusive breastfeeding**			
Yes No	35 (17.5) 165 (82.5)	2 (1.1) 181 (98.9)	37 (9.6) 347 (90.4)
**Infant formula (IF)**			
Yes No	161 (80.5) 39 (19.5)	150 (82) 33 (18)	312 (81.3) 72 (18.8)
Both (BF+IF) (from birth)	37 (18.5)	4 (2.2)	41 (10.6)
**Initiation of infant formula**			
At birth−40 d 40 d−6 months At 4 months >6 months	104 (52.8) 41 (20.8) 14 (7.1) 38 (19.3)	99 (54.1) 43 (23.5) 38 (20.8) 3 (1.6)	203 (52.9) 84 (21.9) 53 (13.8) 41 (10.7)
**Introduction of food**			
At <4 months 4–6 months (6 not included) At 6 months >6 months No food yet	2 (1) 81 (40.5) 55 (27.5) 7 (3.5) 55 (27.5)	3 (1.6) 70 (38.3) 94 (51.4) 16 (8.7) -	5 (1.3) 151 (39.3) 150 (39.1) 23 (6) 55 (14.3)
**Introduction of juices and sugary drinks**			
<4 months 4–6 months 6 months >6 months No food yet	26 (13) 51 (25.5) 56 (28) 14 (7) 53 (26.5)	35 (19.1) 60 (32.8) 72 (39.3) 16 (8.7) -	61 (15.9) 112 (29.2) 128 (33.3) 30 (7.8) 53 (13.8)

*This data was derived from cross-sectional and retrospective recalls*.

#### Children 0–59 Months

The prevalence of EBF, EBOT, MF, and complementary feeding among children ages 0–59 months is shown in Table 2. The prevalence of EBF at 40 d was 27%, at 6 months was 30%, and the prevalence of continued breastfeeding (exceeding 6 months) was 23%. On the other hand, the prevalence of EBOT at birth was 52.9%, at 40 d was 81.3%, at 4 months was 13.8%, and at more than 6 months, it was 10.7%. Moreover, the MF since birth was 10.6%. Around 60% of mothers breastfed their offspring between 0 and 6 months. Half the women introduced infant formula at earlier stages between 0 and 6 months, 78.4% of them introduced food to their children between 4 and 6 months (of which 40% before 6 months), and 62.5% of them introduced sugary drinks before 6 months (Table 2).

#### Household Consumption of Food Groups and Calculation of Mother-Children DDS

Table 2 shows the frequency of consumption of food groups and the DDS among the mothers and their children (6–59 months). There was mother-child agreement in the consumption of staple foods (91.4/91.1%), dairy products (66.7/94.7%), flesh food (74.3/66.8%), and fruits and vegetables poor in vitamin A (74.3/75.7%). Eggs (5/10.5%) and fruits and vegetables that are sources of vitamin A (9.2/16.4%) were the least consumed among food groups. Aged under 5 years, 32% of respondent's children did not get a variety of types of food or provision of food types and were eating less than four kinds of food groups the day before the survey. Similarly, 46% of respondent's mothers were facing a low DDS indicating a poor diversity of food groups at meals (Table 3).

**Table 3:** Frequency of a food group's consumption and dietary diversity score of mothers and children.

	**Children (6–59 months)**		**Mothers**	
**Food groups**	**Consumed *N* (%)**	**Not consumed *N* (%)**	**Consumed *N* (%)**	**Not consumed *N* (%)**
Staples	277 (91.1)	27 (8.9)	277 (91.4)	26 (8.6)
Fruits and vegetables rich in vitamin A	50 (16.4)	254 (83.6)	28 (9.2)	275 (90.8)
Other fruits and vegetables	230 (75.7)	74 (24.3)	225 (74.3)	78 (25.7)
Meat, fish, and poultry	203 (66.8)	101 (33.2)	225 (74.3)	78 (25.7)
Eggs	32 (10.5)	272 (89.5)	15 (5)	288 (95)
Legumes and nuts	88 (28.9)	216 (71.1)	94 (31)	209 (69)
Dairy products	288 (94.7)	16 (5.3)	202 (66.7)	101 (33.3)
	**DDS**		**DDS**	
	High DDS^*^	Low DDS^*^	High DDS^*^	Low DDS^*^
	206 (68)	97 (32)	165 (54.5)	138 (45.5)

* *WHO cutoffs in children: High DDS ≥4 food groups out of 7 consumed, Low DDS <4 food groups out of 7 consumed*.

* *WHO cutoffs in mothers: High DDS≥5 food groups out of 7 consumed, Low DDS <5 food groups out of 7 consumed*.

The association between a child's DDS, socio-demographic characteristics, mother's nutritional status, and child's nutritional status is shown in Table 4. There was a strong correlation between the maternal and offspring's DDS. There were 40% of mothers with low DDS shown to have children with low DDS too (*p*-value = 0.003). Most children with high DDS (75%) had employed mothers (*p*-value = 0.005). Moreover, the children's DDS was related to the mother's education. Mothers holding a university degree had children who were eating an adequate diversified diet (*p*-value = 0.045).

**Table 4:** Association between the child's dietary diversity score and the mother's socio-demographic, education, and nutrition related factors.

	**Low DDS child**	**High DDS child**	**P-value**
	**Count (%)**		
Mother's age categories^*^(***n =*** **303)**			0.23^*^
20–24 25–29 30–34 35–39 40–45	5 (41.7) 27 (37.5) 30 (24.8) 28 (34.1) 7 (43.8)	7 (58.3) 45 (62.5) 91 (75.2) 54 (65.9) 9 (56.3)	
**Mother DDS (*****n =*** **303)**			0.003^*^
Low DDS High DDS	56 (40.6) 41 (24.8)	82 (59.4) 124 (75.2)	
BMI^*^mother (***n =*** **303)**			0.775
Severe underweight Underweight Normal weight Overweight Obese	2 (66.7) 1 (25) 60 (32.1) 22 (31) 12 (31.6)	1 (33.3) 3 (75) 127 (67.9) 49 (69) 26 (68.4)	
**Mother's employment (*****n =*** **303)**			0.005^*^
Employed Unemployed	48 (25.9) 49 (41.5)	137 (74.1) 69 (58.5)	
**Education level (*****n =*** **303)**			0.045^*^
Primary education Intermediate education Secondary education or technical diploma University degree and higher	2 (66.7) 11 (55) 18 (36) 66 (28.7)	1 (33.3) 9 (45) 32 (64) 164 (71.3)	
**Mother's dieting (*****n =*** **303)**			0.969
Yes No	21 (31.8) 76 (32.1)	45 (68.2) 161 (67.9)	
**Children at home < 5 years old (*****n =*** **303)**			0.985
1 2 3 or more	49 (31.6) 42 (32.3) 6 (33.3)	106 (68.4) 88 (67.7) 12 (66.7)	
**Household number (*****n =*** **303)**			0.799
<5 5 or more	58 (32.6) 39 (31.2)	120 (67.4) 86 (68.8)	
**Family income (*****n =*** **303)**			0.000^*^
<750,000 L.L. 750,000–1,500,000 L.L. 1,500,001–2,250,000 L.L. 2,250,001–3,000,000 L.L. >3,000,000 L.L.	4 (80) 23 (48.9) 27 (42.2) 15 (27.3) 28 (21.4)	1 (20) 25 (51.1) 37 57.8) 40 (72.7) 103 (78.6)	

* *BMI, Body mass index*.

* *https://www.who.int/healthinfo/paper31.pdf*.

#### Binary Logistic Regression Analysis

The DD among children (6–59 months) adjusting for covariates at maternal and household levels is described in Table 5. The backward logistic regression showed that the main two factors that are strongly associated with the children's DD are the family income and the mother's DD status. Children's DD increases around 2 times [AOR = 1.7, 95% CI (1.042–2.914)] in context of high maternal DDS, and about 12 times [AOR = 11.7, 95% CI (1.2–111)] when the member of the highest-income households (Table 5).

**Table 5:** Adjusted odds ratio for child dietary diversity score according to family income and the mother's diet diversity score.

	AoR^*^ = Exp (β) (95%CI)	***P*-value**
**Family income**		
<750,000 L.L. 750,000–1,500,000 L.L. 1,500,001–2,250,000 L.L. 2,250,001–3,000,000 L.L. >3,000,000 L.L.	1 3.8 (0.3–36.9) 4.8 (0.4–45.8) 9.5 (0.9–92.8) 11.7 (1.2–111.0)	0.252 0.175 0.054^*^ 0.032^*^
**DDS mother**		0.034^*^
Low DDS High DDS	1 1.7 (1.042–2.914)	

* *Calculated through backward logistic regression model*.

## Discussion

The current study examines the feeding patterns including breastfeeding, formula-feeding, complementary feeding, and the prevalence of malnutrition in under-five children living in two main Lebanese provinces (Beirut and Mount Lebanon). This study also investigates the mother-child's DD and identifies the factors that cause less optimal nutrition due to a lack of food diversity in children aged 6 to 59 months. Our findings demonstrated that in the main two Lebanese provinces, there are low rates of EBF, high rates of EBOT, early initiation of complementary feeding, and early introduction of infant formula among under-two children. These results were reported retrospectively also by mothers of under-five children. Furthermore, for children aged 6–59 months, there was poor mother-child DD, and a high prevalence of overweight and stunted children. In Lebanon, based on a recent report published by UNICEF (21), the early initiation of EBF in 2004 was reported as 41.3% in under-two children. Moreover, according to national data, in 2010, about 40% of infants under 2 months of age were exclusively breastfed, dropping to only 2% between 4–5 months of age (22). In fact, only 37.6% of infants had breast milk as their first food after birth (22). Another national data collected from under-five children's mothers showed that the prevalence of EBF in 2012 at 40 d was 41.5%, which is higher than that reported in our study (27%) (23). Moreover, the same study showed that the prevalence of MF at 40 d was 38.1 and 20.2% were EBOT (23). However, in our study, the MF was 10.6% and the EBOT was 2 times higher (53%). Another recent retrospective cross-sectional Lebanese study published in 2019 showed that the average duration of exclusive breastfeeding was 15 d, while the average age at which formula was introduced was 2.03 months (24). Exclusive breastfeeding began at a mean age of 10.56 h, and half of the toddlers had been exposed to formula milk since the first day after birth (24). In addition, at global and Eastern Mediterranean level, the prevalence of EBF (44 and 44%, respectively) and the early initiation of EBF (48 and 35%, respectively) for children aged under 2 years, between 2014 and 2020 was higher than our findings (26%) (21). This indicates a negative deviation in the pattern of breastfeeding along with an increase in the use of infant formula at birth in Lebanon. All in all, both national, regional, and international prevalence were below the WHO new global target of exclusive breastfeeding (70%) by 2030 (6). The findings of these studies and ours shed light on the lower rate of exclusive breastfeeding and the higher rate of early introduction of formula-feeding, generally in the Eastern Mediterranean Region and specifically in Lebanon. Hence, raising awareness sessions during the pre- and post-natal period and highlighting the successful and vital role of breastfeeding for both the child and the mother are critical.

Regarding complementary feeding, our data found that more than 60% of the mothers introduced complementary foods before the age of 6 months from which 40% started introducing solid foods and 53.5% introduced sugary juices. In our study, only 31% of mothers adhered to the WHO recommendations to introduce foods at 6 months. This finding was below the complementary feeding prevalence reported by UNICEF for the EMR which is 68% and at global level which is 73% (21). However, early introduction of complementary foods and sugary juices have a negative impact on the infant's nutrient intake and may increase the risk of developing non-communicable diseases in later stages (13). Besides, if complementary foods are not handled and stored appropriately, they may expose infants to harmful germs (25). Many factors can be associated with early introduction of solid foods. A previous cross-sectional study conducted in Lebanon showed that women who worked outside the home were nearly twice as likely to start solid foods before the age of 4 months (26). This finding was in line with our findings.

Our current study examined the concordance between maternal and child dietary diversity and factors affecting the concordance. Our study showed that the proportion of discordant is few (*p* = 0.03). This is to mean that the more food groups the mothers consumed, the more likely their children achieved their DD and vice versa. As the mothers' DD increased, the percentage of children (0–59 months) meeting this criterion increased dramatically. Even though there is a dearth of literature on concordance between maternal and child dietary diversity in the EMR and in Lebanon, a related study on maternal and child DD associations in Bangladesh, Vietnam, and Ethiopia showed a fair association between the two (27). This finding is consistent with previous studies conducted in Ghana and South Hampton (28). Mothers belonging to a high-income household and having a high DD score were the two major correlates that lead to an adequate diversified diet among children (6–59 months). This finding came hand in hand with a study published in Nigeria (29), and in line with another study conducted in Madagascar which showed that participants with a low economic status were 1.8 times more likely to have children eating poor diets (30). To explain, lower income households purchase less healthful foods compared with higher income households. Food purchasing patterns may mediate income differences in dietary intake quality. In our study, it was shown that maternal education and employment were correlated with the diversification in mothers and children's diets. This result is consistent with many studies conducted in Ethiopia (31) and Madagascar (30), but not in Nigeria (29). The likely reason might be as the education level of the mother increased and as the mother engaged in paid work, there was access to more information on educational messages and different mass media like radio, television, and newspapers. They also participate actively in health education sessions and child feeding demonstrations in health facilities; as a result, their children are more likely to fulfill the DD requirement. In this study, on average, three food groups composed the diets of under-five Lebanese children, which are staples (grains/cereals, roots, and tubers) (91%), dairy products (milk, yogurt, and cheese) (94.7%), and fruits and vegetables (76%), whereas the least two food groups consumed were eggs (10.5%) and fruits and vegetables rich in vitamin A (16.4%). Consequently, one out of two mothers and one out of three children (6–59 months) had a low DDS (46 and 32%, respectively). Our findings were found to be consistent with different studies such as Pakistan (75.2% for staples and 50.5% for the dairy products) (32). In Nigeria, the most consumed two food groups are cereals (0.78 ± 0.29) and vegetables (0.78 ± 0.3), while the least consumed food group is eggs (0.15 ± 0.25) (29). In Ethiopia, the consumption of grains and staples is 85%, that of eggs is 90%, and that of vitamin A rich fruits and vegetables is 80% (27). As for Vietnam, food consumption of staples was 97% and eggs were 75%, and Bangladesh's staple consumption was 90 and 80% for eggs (27).

As for malnutrition, most of the children aged under 5 in this study had a normal body weight (44%). In addition, 9.3% were underweight (WAZ < -2SD to −3SD), 6.5% were at risk of being overweight, 24.45% were overweight, 9.3% were stunted (HAZ < -2SD to −3SD), and 6.25% (WHZ < -2 SD to −3SD) were wasted. The prevalence of stunting, wasting, and overweight among under-two children was 12.5, 37.5, and 62.5%, respectively. According to WHO, in 2020, globally, 149.2 million children under the age of 5 years were stunted (22%), 45.4 million wasting (13.6%), and 38.9 million overweight (5.7%), respectively (3). According to the UNICEF database, published in 2021, the global prevalence of stunting among under-five children ranged between 33.9% in 2000 and 27.9% in 2020 (33). Moreover, the prevalence of overweight among children aged 0–59 months ranged between 33.3 and 42.4% between 2000 and 2020 (34). Also, the prevalence of wasting in 2020 was 13.6% (34). At the EMR level, according to a new published review, stunting, wasting, and underweight had an average prevalence of 28, 8.69, and 18%, respectively (35). In Lebanon, the prevalence of stunting ranged between 16% in 2000 to 10.4% in 2020 (5). As for overweight and obesity, in 2018, the estimated weighted average prevalence in children aged under 5 years was 8.42% in the EMR and between 17.6 and 19.7% in Lebanon (5). For the prevalence of wasting in Lebanon and Jordan, national surveys conducted in 2012 showed that the prevalence of stunting ranged between 7.3 and 7.7% (36, 37), between 1.1 and 2.4% for wasting, and between 1.6 and 3% for underweight (36, 38). The annual rates of change in the prevalence of stunting were estimated to be −6.9 % in Lebanon (2004–2012) and −3.3% in Jordan (2002–2012). The prevalence of wasting was found to be stable over time in Jordan (2.4% in 2002 and 2012) and decreasing in Lebanon (from 6.6% in 2004 to 1.1% in 2012) (39). All in all, our findings were in line with UNICEF's data in 2020 concerning stunting's prevalence (5), were lower than the EMR's data and the UNICEF's data regarding wasting's prevalence (34, 35), were higher than the previously published national data (5) and lower than the UNICEF data concerning the prevalence of overweight (5, 34).

## Limitations and Strengths

The study offers a unique lens into infant feeding practices related to under-five children in Lebanon. However, it has several limitations. First, it is a cross-sectional study with unclear chronology of the factors associated with infant feeding patterns. Second, mothers reported infant feeding practices of their children, with potential for more recall bias among mothers of older children due to a longer time since delivery; however, almost half of the children were younger than 5 years of age.

## Conclusion and Future Perspectives

Our findings demonstrated low rates of EBF and continuous BF, high use of IF, and early introduction of complementary feeding among children aged 0–23 months. Furthermore, for children 6–59 months, there was poor mother/child DD, and a high prevalence of overweight and stunted children in the main two Lebanese provinces. This suggests the alarming need for continuous nutrition intervention to improve the DD and to reduce the malnutrition rates. Future research should investigate the factors associated with EBF, complementary feeding practices, and DD in prospective cohort studies and help to better understand the cultural factors limiting the adherence to international guidelines. Such research can guide effective planning for interventions to improve infant feeding practices and ultimately children's health status.

## Data Availability Statement

The original contributions presented in the study are included in the article/supplementary material, further inquiries can be directed to the corresponding author.

## Ethics Statement

The studies involving human participants were reviewed and approved by Ethical Committee Lebanese University. The patients/participants provided their written informed consent to participate in this study.

## Author Contributions

MHo: conceptualization, methodology, supervision, and writing—reviewing and editing. HA and MHa: data curation, writing—original draft preparation, visualization, and investigation. All authors contributed to the article and approved the submitted version.

## Conflict of Interest

The authors declare that the research was conducted in the absence of any commercial or financial relationships that could be construed as a potential conflict ofinterest.

## Publisher's Note

All claims expressed in this article are solely those of the authors and do not necessarily represent those of their affiliated organizations, or those of the publisher, the editors and the reviewers. Any product that may be evaluated in this article, or claim that may be made by its manufacturer, is not guaranteed or endorsed by the publisher.
